# Accumulation of Temozolomide-Induced Apoptosis, Senescence and DNA Damage by Metronomic Dose Schedule: A Proof-of-Principle Study with Glioblastoma Cells

**DOI:** 10.3390/cancers13246287

**Published:** 2021-12-14

**Authors:** Lea Beltzig, Björn Stratenwerth, Bernd Kaina

**Affiliations:** Institute of Toxicology, University Medical Center, D-55131 Mainz, Germany; lea.beltzig@uni-mainz.de (L.B.); b.stratenwerth221@gmail.com (B.S.)

**Keywords:** temozolomide, apoptosis, senescence, dose schedule, metronomic doses, glioblastoma

## Abstract

**Simple Summary:**

Severe toxic side effects do not allow unlimited dose escalation of anticancer drugs, and the doses used in cancer therapy are therefore often rather low regarding the required target concentration. For temozolomide (TMZ), which is used in glioblastoma therapy, single high dose protocols are used in nearly all experimental studies, while the drug is administered repeatedly on patients, with a daily (metronomic) low dose schedule. Here, we show that the therapeutically relevant glioblastoma cell death and senescence responses do accumulate if a high dose of TMZ is split up in small low doses. The data support the metronomic dose schedule and suggest that even low doses are effective in glioblastoma therapy. The predominance and accumulation of TMZ-refractory senescent survivors may provide an explanation for the overall low curative response.

**Abstract:**

Temozolomide (TMZ), a first-line drug in glioma therapy, targets the tumor DNA at various sites. One of the DNA alkylation products is *O^6^*-methylguanine (*O^6^*MeG), which is, in the low dose range of TMZ, responsible for nearly all genotoxic and cytotoxic effects relevant for cancer therapy. There is, however, a dispute regarding whether the TMZ concentration in the tumor tissue in patients is sufficient to elicit a significant cytotoxic or cytostatic response. Although treatment with TMZ occurs repeatedly with daily doses (metronomic dose schedule) and in view of the short half-life of the drug it is unclear whether doses are accumulating. Here, we addressed the question whether repeated low doses elicit similar effects in glioblastoma cells than a high cumulative dose. We show that repeated treatments with a low dose of TMZ (5 × 5 µM) caused an accumulation of cytotoxicity through apoptosis, cytostasis through cellular senescence, and DNA double-strand breaks, which was similar to the responses induced by a single cumulative dose of 25 µM TMZ. This finding, together with the previously reported linear dose–response curves, support the notion that TMZ is able to trigger a significant cytotoxic and cytostatic effect in vivo if the low-dose metronomic schedule is applied.

## 1. Introduction

Temozolomide (TMZ, Temodal^®^, Temodar^®^) is a DNA-methylating agent frequently used in cancer therapy [1]. It is applied first-line for high-grade gliomas, notably glioblastoma multiforme (astrocytoma WHO grade 4, GBM) [2] and for some other cancers [3]. Despite dissection of the tumor after diagnosis and radiochemotherapy, patients have a dismal prognosis with, on average, 14.6 months (12.6 and 23.4 months in the *O^6^*-methylguanine-DNA methyltransferase (MGMT)-unmethylated and MGMT-methylated sub-group, respectively) [4] and a 2- and 5-year survival of 26.5% [2] and <10% [5], respectively. TMZ is effective if the tumor lacks MGMT [6] or expresses the repair protein at a low level [7], supporting the notion that MGMT is an important prognostic marker [8].

The low curative response has raised the question of the cytotoxic potency of TMZ. Moreover, in experimental settings, doses of TMZ are often applied that are above the level that can be achieved in vivo, i.e., >100 µM [9,10]. In some studies, even millimolar concentrations of TMZ were used [11]. It is clear that the data obtained under these experimental conditions can hardly be translated to the in vivo situation, where intratumoral TMZ concentrations between 1 and 35 µM are achieved (see discussion). However, there is also evidence that low doses of TMZ (2–50 µM) elicit an apoptotic response, which can be measured if cells have passed through two or more cell cycles after treatment [12]. Since the observed cytotoxic responses were low (up to 20% apoptosis), the results further fueled the assumption that TMZ is ineffective as a cytotoxic agent [13].

In the therapeutic setting, after tumor resection, TMZ is used concomitantly with focal radiotherapy, followed by adjuvant TMZ [4]. As a protocol example, within 6 weeks, single doses of 2 Gy (total 60 Gy) are co-administered with TMZ (75 mg/m^2^ per day), which are followed by 6 TMZ cycles (5 days/week, 150–200 mg/m^2^ daily) [2]. TMZ is also used in recurrent glioblastoma. Here, the most frequent schedules are the continuous daily administration [14,15,16], the “3 weeks on/1 week off” [17] or the “1 week on/1 week off” protocol [18]. In all protocols, TMZ is administered daily with low, tolerable doses. Even long-term treatment is well tolerable [16]. In view of the treatment regimens, the question arises as to whether the effects of small doses of the drug accumulate, intensifying the cytotoxic effects.

We tested this question experimentally. We treated cells with a single high dose (25 µM), which is at the upper level in the linear dose range [19] and approximates the achievable serum concentration [20], and split it up in several small doses (5 µM each) and measured the ultimate critical damage DNA double-strand breaks (DSB), apoptosis and induced cellular senescence (CSEN). The data revealed a significant low-dose accumulation of these effects, approaching the yield of a single high dose of these measured endpoints

## 2. Materials and Methods

### 2.1. Cell Lines and Culture Conditions

The human glioblastoma cell lines LN229 and A172 were purchased from American Type Culture Collection (ATCC). Their properties were described previously [21,22]. Cells were cultured in DMEM with Glutamax (Gibco, Life Technologies Corporation) and 10% fetal calf serum at 37 °C in a humidified 5% CO^2^ atmosphere. Cells were treated 24–48 h after seeding when they were in the exponential growth phase. For short-term experiments (harvest 3 d after treatment), 2 × 10^5^ cells were seeded per 5-cm or 6-well dishes, and for long-term experiments (apoptosis and senescence, harvested 5 and 10 d following TMZ treatment, respectively), 10^5^ cells were seeded per dish. For long-term experiments, cells were split and reseeded 6 d after the first treatment. Cells were kept in exponential growth for the whole experimental period until they died or became senescent.

### 2.2. Drugs and Drug Treatment

TMZ obtained from Dr. Geoff Margison (University of Manchester, Manchester, UK) was dissolved in DMSO (150 mM stock) and stored in 50 µL batches at −80 °C until use. Immediately before treatment, the stock was diluted 1:10 in sterile distilled water and added to the cell culture medium at the desired final concentration. For the lower concentrations (≤10 µM), the solution was further diluted with distilled water to a 1 mM stock. The amount of DMSO in the medium did not exceed 0.05% and was without any toxic effect (controls).

### 2.3. Quantification of Apoptosis

The fraction of apoptotic and late apoptotic/necrotic cells was determined by flow cytometry 5 d after the last treatment using annexin V/FITC and propidium iodide (A/PI) staining of cells [23]. In brief, cells in the supernatant and trypsinized cells were collected, washed with PBS, and stored on ice. Cells were incubated for 15 min in the dark in 50 µL annexin binding buffer containing 2.5 µL annexin V/FITC (Miltenyi Biotec GmbH, Bergisch Gladbach, Germany). For PI staining, 10 µL propidium iodide from a 50 µg/mL stock solution (Sigma-Aldrich, Steinheim, Germany) were added to each sample. Cells were incubated for additional 10 min on ice and kept in the dark until measurement using a FACS Canto II flow cytometer (Becton Dickinson GmbH, Heidelberg, Germany). Data was analyzed using the Flowing Software 2 program (Perttu Terho, Turku Center for Biotechnology, University of Turku, Turku, Finland). Apoptotic cells were defined as annexin V+/PI−, whereas late apoptotic/necrotic cells were defined as annexin V+/PI+ cells (for representative plots see [23]).

### 2.4. Measurement of Induced Cellular Senescence

TMZ-induced cellular senescence (CSEN) was determined 8 d after the last treatment via senescence associated β-galactosidase (ß-Gal) and flow cytometry. To inhibit endogenous β-Gal activity, cells were preincubated with 300 µM chloroquine for 30 min at 37 °C. Thereafter, C12FDG was added to each sample (final concentration 33 µM). After 90 min incubation, cells were washed with cold PBS and collected by trypsinization. Cell pellets were washed, resuspended in cold PBS, and stored on ice. After addition of chloroquine they were kept in the dark up to harvest. Data acquisition was performed using FACS Canto II flow cytometer and the Flowing Software 2 program (see above, Section 2.3.). Untreated, proliferating cells were used as the control. Cells with a fluorescence intensity higher than the control were defined as senescent (for representative plots see [23]).

### 2.5. Measurement of γH2AX and 53BP1 Foci

The γH2AX and 53BP1 foci assay was performed essentially as described [23]. Cells were grown on coverslips and fixed 3 d after the last treatment. The evaluation of γH2AX and 53BP1 foci occurred by LSM. At least 100 cells were measured per experiment. Antibodies used were γH2AX (1:500, rabbit, Cell Signaling Technology, mAb #9718S) combined with Cy3 goat-anti-rabbit (1:500, Abcam, ab97075) and 53BP1 (1:500, mouse, Sigmar Aldrich, MAB 3802) combined with Alexa Fluor 488 goat-anti-mouse (1:500, Thermo Fisher Scientific Invitrogen, A11017). Foci were counted by means of the software ImageJ (Wayne Rasband, NIH).

## 3. Results

**Figure 1:**
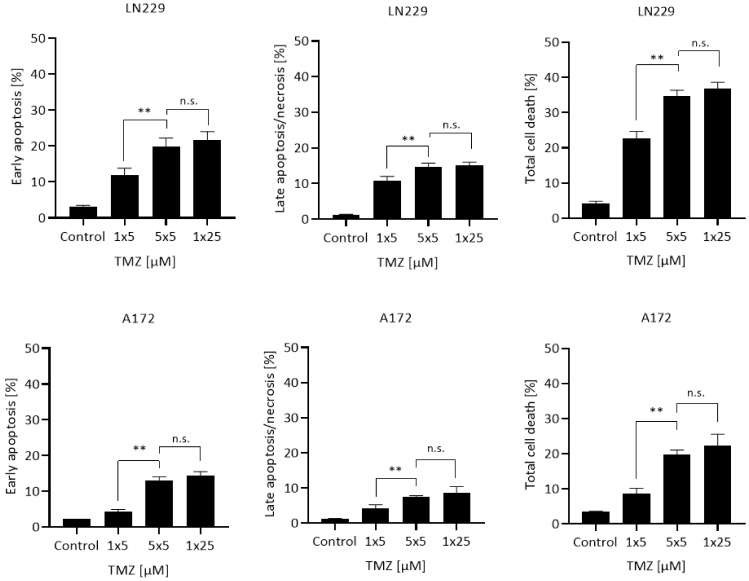
Apoptosis, late apoptosis/necrosis, and total cell death induced in LN229 and A172 cells following treatment with a single dose of 5 µM (1 × 5), repeated doses of 5 µM (5 × 5), and a single cumulative dose of 25 µM (1 × 25). Data are presented as mean of three independent experiments with triplets measured ±SEM and compared by unpaired *t*-test with Welch’s correction. n.s. = not significant; ** *p* < 0.01.

First, we addressed the question of whether repeated treatment with a low dose causes an accumulation of cytotoxic effects. To this end, the glioblastoma cell lines LN229 and A172 were treated with a single dose of 5 µM, 5 repeated doses of 5 µM, and a cumulative single dose of 25 µM. Similar to the patient’s treatment schedule, the split doses were administered in daily (24 h) intervals. The results shown in Figure 1 demonstrate that early apoptosis, late apoptosis/necrosis, and the total cell death level were significantly higher in the repeated dose protocol compared to a single low dose. Interestingly, a cumulative dose of 5 × 5 µM results in the same effect that was measured following treatment with a single high dose of 25 µM. The data revealed that repeated low doses of an alkylating agent in MGMT deficient cells give rise to an accumulation of the toxic effects brought about by each single dose.

**Figure 2:**
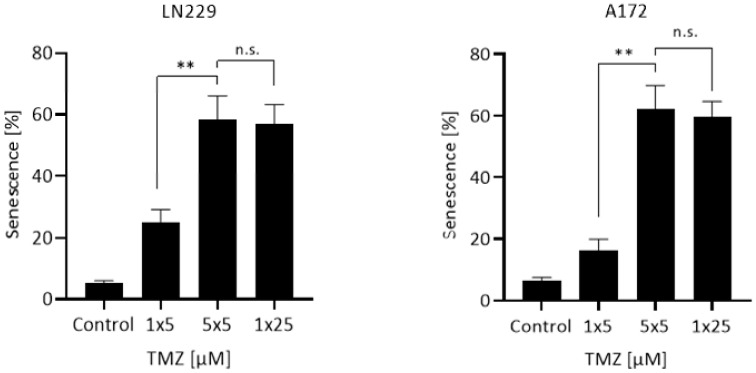
CSEN induced in LN229 and A172 cells following treatment with a single dose of 5 µM, repeated doses of 5 µM, and a single dose of 25 µM. Data are presented as mean of three independent experiments with triplets measured ±SEM and compared by unpaired *t*-test with Welch’s correction. n.s. = not significant; ** *p* < 0.01.

Similar experiments were performed measuring the endpoint CSEN. LN229 and A172 cells were again treated with the patient’s treatment schedule of 5 daily doses, 5 µM each, yielding a cumulative dose of 25 µM, or a single dose of 5 or 25 µM. The results shown in Figure 2 revealed that repeated low doses of TMZ induce a similar effect than a single high dose. Obviously, apoptosis and CSEN accumulate in a metronomic dose schedule.

**Figure 3:**
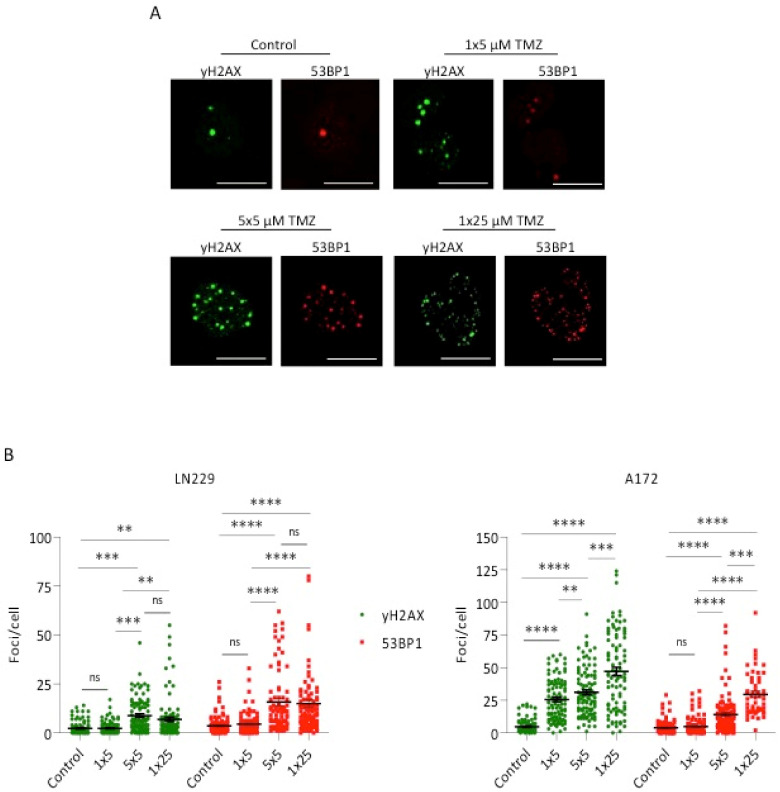
Induction of DSBs, measured by γH2AX and 53BP1 staining and foci quantification through LSM, in LN229 and A172 following a single dose of 5 or 25 µM or 5 repeated doses, each of 5 µM TMZ. (**A**) Representative images for γH2AX and 53BP1 foci staining in LN229 cells for the indicated treatments. Scale bars indicate 30 µm. (**B**) Foci number per cell in the controls and upon treatment. Data of a representative experiment are shown, with 100 cells counted for each treatment. Bars indicate the mean. n.s. = not significant; ** *p* < 0.01; *** *p* < 0.001; **** *p* < 0.0001.

The study was extended measuring the level of DNA double-strand breaks (DSB) by γH2AX and 53BP1 foci quantification [19]. Representative images are shown in Figure 3A. As shown in Figure 3B, a single dose of 25 µM enhanced the level of γH2AX and 53BP1 significantly above the control level. This was also the case if cells were treated with 5 × 5 µM TMZ, which was more effective than a single dose of 5 µM. It should be noted that the cumulative effect of 5 × 5 µM TMZ was not as strong as observed for the endpoint apoptosis and CSEN, which is presumably due to repair of DSBs occurring in the period between the single doses. Therefore, a higher outcome of DSBs can be anticipated if a single high dose is applied, which is indeed the case. Nevertheless, the data revealed that repeated treatments with low doses give rise to a significantly enhanced yield of DSBs, which approached the effect of a single high dose.

## 4. Discussion

In nearly all in vitro experiments, the effect of the DNA methylating drug TMZ is investigated following a single dose administration. In the therapeutic setting, however, TMZ is given daily, according to different schedules [2,16,18,24]. In some protocols, treatment occurs over a period of several weeks, according to a metronomic (dose-dense) schedule [16,25]. Here, we demonstrate in cell culture experiments, which allow precise dosing and quantitative measurements, that the metronomic low dose application is similar effective than a single high dose protocol. It is important to note that for these experiments, glioblastoma cells that lack MGMT were used [21]. Therefore, the critical lesion *O^6^*MeG is expected to accumulate and be processed in the post-treatment cell cycles by MMR to give rise to DSBs that trigger downstream cell death and senescence pathways [12,26]. In MGMT expressing cells, which are highly resistant to TMZ, cumulative low doses are likely ineffective because the critical lesion is repaired after each single cycle. It should be noted, however, that MGMT becomes inactivated during the repair process and, therefore, the metronomic dose schedule might exert some effect also in MGMT positive cells if the MGMT expression level is sufficiently low. Interestingly, a threshold level of 30 fmol MGMT was identified below which the therapeutic effect was significant [7], which supports this view.

The serum half-life of TMZ is about 2 h and the serum concentration of TMZ was shown to be in the range of 20 up to 70 µM [1,27,28,29,30,31]. In more detail, administering an oral dose of 150 mg/m^2^, the peak plasma concentration was, on average, 28.4 µM (5.5 µg/mL) and the brain interstitium and peritumoral concentration was 3.2 µM (0.6 µg/mL) [20]. In another study using oral 200 mg/m^2^ TMZ, the plasma peak level was 72 µM and the concentration in the cerebrospinal fluid was 9.9 µM [32]. The TMZ concentration in treated patients was also measured by positron emission tomography (PET). Following treatment with 75–200 mg/m^2^/d, intratumoral peak concentrations of 14.9–34.5 µM were measured, which were even higher than the concentrations in the normal brain [33]. Using the U87 rat glioma model and microdialysis method, an intratumoral maximal concentration of 3 µM was measured [34]. It is important to note that TMZ can pass the blood–brain barrier and thus reaches the brain unhindered. There are also no transporters involved that could be dose-limiting. Taken the available data together, the intratumoral TMZ concentration is likely in the range of 3–35 µM. In vitro experiments showed that even in this low dose range cytotoxic effects are induced [19,23]. Thus, we showed in LN229 and A172 cells that the amount of *O^6^*MeG in the DNA and the number of DSBs (as measured by γH2AX and 53BP1 foci formation) increase linearly with dose of TMZ [23]. The same was found for apoptosis and senescence. We did not observe a significant threshold for critical primary DNA lesions, apoptosis, and senescence in this cell system [19,23].

The findings support the notion that low doses of TMZ are effective, provided that the tumor cells are MGMT lacking [35]. The accumulation of several single low dose effects to a level nearly comparable to a single cumulative high dose provides a mechanistic basis for the observation that TMZ administered according to the metronomic dose schedule is effective. This dose schedule has the advantage that toxic side effects are reduced since they allow low level expressing normal cells, such as hematopoietic CD34 stem cells [36], to survive through repair of *O^6^*MeG.

We should stress the point that low TMZ doses induce apoptosis, although senescence is the predominant trait, which applies to single dose and metronomic treatment conditions. Obviously, TMZ bears both cytotoxic and cytostatic activity. The high yield of TMZ-induced senescent cells might provide an explanation for the low curative response since senescent cells are long-term survivors [37] and it cannot be ruled out that they will be reactivated to proliferation leading to recurrences.

We are aware that the study is limited by the fact that cells were examined in vitro. Both cell lines are p53 functionally wild-type. Therefore it remains to be clarified whether p53-mutated glioma cells, which prefer the mitochondrial apoptosis pathway [38] respond in the same way. The question of whether the data can be transferred to the in vivo situation is also open. The effect of metronomic doses is difficult to study in patients, as samples would have to be taken repeatedly during therapy in order to examine DNA damage, senescence, and apoptosis markers. However, corresponding experiments on tumor-bearing animals should be feasible and are warranted on the basis of this data.

## 5. Conclusions

Toxic side effects do not allow unlimited dose escalation of anticancer drugs. Therefore, the administered therapeutic doses are often rather low and thought to be ineffective. TMZ is well tolerated and administered repeatedly on patients, with a metronomic schedule. The finding reported here that repeated low-dose treatments lead to an accumulation of toxic and cytostatic effects supports the view that repetitive treatments with low doses of TMZ (metronomic dose schedule) are effective and represent a reasonable treatment strategy. Overall, the data support metronomic (dose-dense) TMZ administration and suggest that even low doses are effective in glioblastoma therapy, which applies at least to promoter methylated MGMT-lacking tumors.

## Data Availability

The data presented in this study are available in article.
